# Serum Pentraxin 3 as Promising Biomarker for the Long-Lasting Inflammatory Response of COVID-19

**DOI:** 10.3390/ijms241814195

**Published:** 2023-09-17

**Authors:** Anna Paola Capra, Lelio Crupi, Giuseppe Pantò, Alberto Repici, Fabrizio Calapai, Raffaele Squeri, Alessio Ardizzone, Emanuela Esposito

**Affiliations:** 1 Department of Chemical, Biological, Pharmaceutical and Environmental Sciences, University of Messina, Viale Ferdinando Stagno d’Alcontres, 98166 Messina, Italy; annapaola.capra@unime.it (A.P.C.); lelio.crupi@studenti.unime.it (L.C.); alberto.repici@studenti.unime.it (A.R.); fabrizio.calapai@unime.it (F.C.); eesposito@unime.it (E.E.); 2 Department of Biomedical and Dental Sciences and Morphofunctional Imaging, University of Messina, Via Consolare Valeria 1, 98125 Messina, Italy; peppepanto@hotmail.it (G.P.); raffaele.squeri@unime.it (R.S.); 3 Department of Clinical and Experimental Medicine, University of Messina, Via Consolare Valeria 1, 98125 Messina, Italy

**Keywords:** Pentraxin 3 (PTX3), inflammation, cytokine storm, immunopathology, COVID-19, SARS-CoV-2

## Abstract

Currently, biological markers for COVID-19 disease severity still constitute the main goal of enhancing an efficient treatment to reduce critical consequences such as an abnormal systemic inflammatory response. In this regard, the latest research has shown that Pentraxin 3 (PTX3), a highly conserved innate immunity protein, may serve as a valuable biochemical marker. Based on this evidence, we conducted a case–control study to compare the PTX3 serum levels and several immune-inflammatory mediators of 80 healthcare workers who were subdivided into subjects who were previously infected with SARS-CoV-2 (*n* = 40) and individuals who were never infected (*n* = 40). Using a commercially available Enzyme-Linked Immunosorbent Assay (ELISA), PTX3 and various immune-inflammatory protein levels were assessed in serum samples, while also considering possible variables (e.g., gender-related differences). We have shown elevated levels of PTX3 and other inflammatory proteins in previously infected COVID-19-positive subjects (*p* < 0.001). Moreover, the obtained data also indicate a degree of severity influenced by gender, as shown by the subgroup analysis, in which PTX3 expression was more pronounced in previously COVID-19-positive males (*p* < 0.001) than in females (*p* < 0.05) compared to the respective controls. In addition, our data further validate, through a direct comparison of previously COVID-19-positive subjects, greater pro-inflammatory levels in males than in females. Overall, our results may support the validity of PTX3 as a systemic biomarker in prolonged systemic inflammatory responses in the context of COVID-19. Thus, PTX3 modulation could constitute an effective therapeutic strategy for improving the recovery from COVID-19 and its systemic long-term consequences.

## 1. Introduction

Since late 2019, the coronavirus disease (COVID-19) has spread globally, determining unprecedented consequences on health, social, and economic systems worldwide. Currently, according to the WHO data, as of June 2023, it has caused more than 6.9 million deaths [1].

After an incubation period of around six days, the disease may become symptomatic in the affected patient, with an extremely wide degree of severity [2,3]. Indeed, it can be either entirely asymptomatic in about 40% of patients, or manifest with a variety of symptoms that may range from mild to critical [4].

In response to SARS-CoV-2 infection, the human organism undergoes two distinct responses. The first phase is the innate immune response, which serves as the first line of defense for the immune system. Over this phase of the process, innate immune cells produce interferons and cytokines, which trigger the adaptive immune response. Virus-infected cells are targeted by CD8^+^ cytotoxic T-cells, whereas CD4^+^ helper T-cells activate B-cells for the production of antibodies that are directed against antigens expressed by the virus (i.e., S protein and RBD) [5].

In this frame, the overwhelming inflammation observed in some COVID-19 patients who developed severe illness has become one of the virus’s defining hallmarks [6]. An abnormal immune response mediated by a variety of cytokines contributes to the progression and related detrimental effects induced by the disease, leading to a “cytokine storm” and the consequent mobilization of neutrophils, macrophages, and T-cells from the blood circulating into the infected tissues [7].

Because of this, COVID-19 patients can continue to experience symptoms and clinical consequences, defined as a whole as “long COVID-19”, for weeks or even months after the acute phase of the disease [8]. This condition, also defined as “post-acute COVID-19 syndrome” (PCOD), usually affects patients regardless of their viral status, as symptoms are usually quite present in PCR-negative subjects with a history of past disease [8]. More than 50 symptoms have been directly linked to long COVID-19, which is highly heterogeneous within the population. Of these, fatigue, which affects about 58% of patients, is the most common symptom, followed by headaches, attention disorders, olfactory dysfunctions, and dyspnea [9]. In addition, the symptoms of long-term COVID-19 (“long COVID”) may also affect organs and systems, ranging from neurological ones causing brain fog, cognitive disorders, sleep disturbances and vestibular symptoms to cardiopulmonary ones triggering palpitations, chest pains and coughing [10]. Nevertheless, in this attractive area of research, the mechanisms underlying long COVID-19 symptoms and their pathophysiology are still largely unknown. Although they seemingly have complex and multifaceted causes, which seem to involve organ damage, coagulation dysfunction, and immune deregulation, most of them are associated with a chronic alteration of inflammatory processes [11].

Several studies demonstrated that a prolonged pro-inflammatory state, which is characterized by the increase in inflammatory markers, such as IL-1β, IL-6, and TNF-α, is a distinctive feature of patients with long COVID-19 [12] since these inflammatory alterations have been shown to persist in some patients for several months after infection, leading to a condition clinically known as post-COVID-19 multisystem inflammatory syndrome (MIS) or a systemic inflammatory response (SIR) [13,14].

Despite several advances made in the past three years regarding the pathophysiology of the acute phase of the disease, several unclear elements remain regarding the long-term inflammatory responses induced by a SARS-CoV-2 infection [10]. Thus, in this clinical context, the identification of reliable new biomarkers to be used as diagnostic tools or predictors of MIS or SIR still represents an unmet medical need in the COVID-19 field.

Since alterations in immune cell levels and a relevant increase in inflammation markers are more prevalent among individuals with severe COVID-19, the acute inflammatory response has been widely studied, allowing for the identification of several factors and serum levels, which significantly change during the COVID-19 inflammatory response, and are likely correlated with the severity of the clinical course [15]. For example, several early studies comparing the serum expression levels of a range of cytokines and other inflammatory factors detected higher levels of IL-2, IL-7, IL-8, IL-10, TNF-α, GSCF, IP10, MCP1, and MIP1A, among others, in intensive care patients’ plasma compared to non-ICU patients [16,17]. In this regard, it has also gradually become more evident how Pentraxin-3 (PTX3) may represent a relevant prognostic marker and a potential predictor of the clinical outcome of a COVID-19 infection [18,19]. PTX3 can be quickly released by both a variety of tissue cells and serum leukocytes, including fibroblasts, endothelial cells, monocytes, macrophages, and dendritic cells in response to several stimuli, such as IL-1 and TNF-α, PAMPs (LPS, lipoarabinomannan, and Omp) and HDL [20,21]. PTX3 differs from the short pentraxins, CRP and SAP, which are synthesized in response to interleukin IL-6 by the liver [22]. Indeed, a significant increase in PTX3 levels can be observed as soon as 6 h after a pro-inflammatory insult, while CRP levels take about 24–30 h to rise [23].

Moreover, as a vital component of humoral innate immunity, PTX3 is involved in a range of physiological processes, like inflammation, complement pathway modulation, immune cell recruitment, and tissue repair [24]. Nevertheless, in contrast to a physiological function, PTX3 seems to be involved in the progression of pathological inflammatory processes [25].

Once released, PTX3 affects a variety of different receptors involved in various aspects of the inflammatory response such as tissue repair processes and angiogenesis [26]. Focusing specifically on its role in the immune response and inflammation, one of the best-characterized effector mechanisms of PTX3 is represented by its multifaceted interactions with components of different complement pathways [27].

It has indeed been demonstrated that PTX3’s ability to regulate both the classical complement cascade by recognizing and binding the complement component 1q (C1q). As well, PTX3 interacts with the lectin pathway by networking with ficolin-1, ficolin-2, and the Mannose-Binding Lectin (MBL) [27]. As well as the activation of the complement system, many other receptors involved in the immune response have been identified in recent years. Among these, scientific evidence suggests that PTX3 is directly able to up-regulate the TLR4-mediated NF-kB signaling, a key pathway in the induction of several pro-inflammatory genes and the secretion of inflammatory mediators [28]. Finally, it was shown that PTX3 can interact with the adhesion molecule P-selectin through its glycosidic domain, thus also regulating the recruitment of inflammatory cells [29].

Furthermore, according to a complement-mediated mechanism, PTX3 can stimulate neutrophil phagocytic activity consequently increasing and prolonging the inflammatory response [23].

In particular, it has been observed that PTX3 is involved and markedly overexpressed in uncontrolled pathological inflammatory responses like the “systemic inflammatory response syndrome” (SIRS), a severe, dysfunctional, and excessive reaction to noxious stimuli that may ultimately result in sepsis. For all the above, since excessive innate immune response is one of the most important hallmarks of severe COVID-19, PTX3 quantification has been considered a potential prognostic tool to identify critical outcomes of high-risk patients [30].

We previously demonstrated, in our systematic review and meta-analysis, that PTX3 levels are significantly associated with disease severity and mortality [31]. In particular, the expression of this inflammatory marker was markedly increased in intensive care unit (ICU) hospitalized patients compared to non-intensive wards patients [31].

On these bases, considering the pivotal role of PTX3 following COVID-19 infection, we conducted a retrospective cohort study evaluating a potential correlation between PTX3 serum levels and systemic inflammatory reaction within a group of hospital workers.

Furthermore, to assess variances in the inflammatory response across subpopulations, we also performed a subgroup analysis evaluating gender-related differences. Therefore, from this perspective, this study aimed to provide new insights into the pathophysiology of COVID-19-related systemic inflammatory response (SIR) while exploring PTX3-driven inflammatory response in the COVID-19 clinical setting.

## 2. Results

### 2.1. PTX3 Sustained Inflammation in SARS-CoV-2 Positive Subjects Compared to Never-Infected Individuals

PTX3 is a crucial mediator of inflammation and immunity, and it is especially increased in response to initial pro-inflammatory signals linked to TLR activation, pathogens, and viral infections as well as following tissue injury [19]. Thus, considering the key role of this protein in COVID-19 pathogenesis, we assessed its levels in human serum using an ELISA kit.

**Figure 1:**
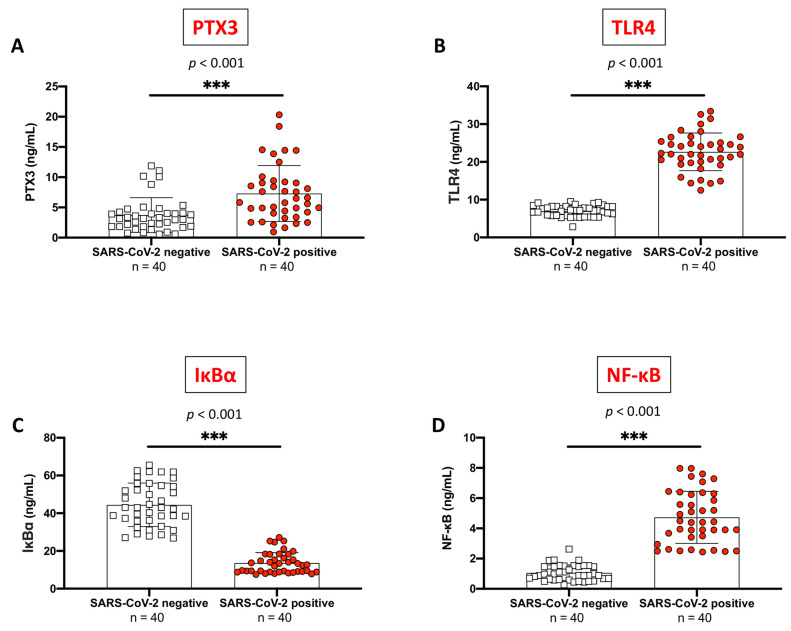
Serum PTX3, TLR4, IκB-α, and NF-κB levels in SARS-CoV-2 positive subjects (*n* = 40) and never-infected individuals (*n* = 40). PTX3 (**A**), TLR4 (**B**), and NF-κB (**D**) serum levels were significantly higher in “SARS-CoV-2 positive” compared to “SARS-CoV-2 negative” (*p* < 0.001). Conversely, IκB-α (**C**) serum levels were significantly lower in “SARS-CoV-2 positive” individuals than in never-infected subjects (*p* < 0.001). White squares indicate SARS-CoV-2 negative values, and red circles indicate SARS-CoV-2 positive values. All data are shown as means ± SD. Statistical differences between means were calculated using the unpaired *t*-test with Welch’s correction. *** *p* < 0.001 vs. “SARS-CoV-2 negative”.

From our analyses, we found that PTX3 levels were significantly higher in SARS-CoV-2 positive subjects compared to never-infected individuals (Figure 1A, *p* < 0.001). 

Among the many TLRs, TLR4 activation is mostly brought about by direct contact with the spike protein SARS-CoV-2, thus triggering macrophage activation and a subsequent inflammatory cascade [32] driven by NF-κB pathway promotion [33].

Our data highlighted a noteworthy hyperactivation of TLR4 expression in SARS-CoV-2 positive individuals compared to SARS-CoV-2 negative (Figure 1B, *p* < 0.001). Consequently, TLR4 overexpression augmented inflammatory responses. Indeed, SARS-CoV-2-infected subjects revealed a notable upturn in NF-κB expression and increased cytosolic degradation of IκB-α compared to the SARS-CoV-2 negative (Figure 1C,D, respectively, *p* < 0.001).

### 2.2. Evaluation of Pro-Inflammatory Cytokines Profile between SARS-CoV-2 Positive Subjects Compared to Non-Infected Control Individuals

It has been extensively demonstrated that the inflammatory reaction linked with COVID-19 disease led to the cytokine storm phenomenon, which induces the production of TNF-α, IL-1β, and IL-6. Therefore, we assessed this cytokine profile in serum samples by employing the ELISA method to verify the inflammatory state after COVID-19 infection.

**Figure 2:**
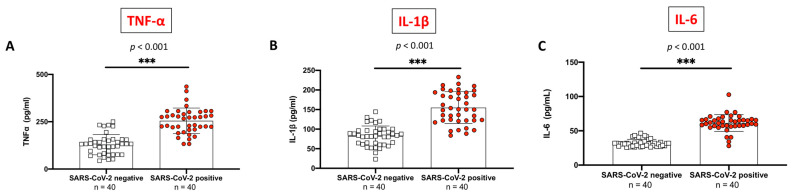
Serum levels of the pro-inflammatory cytokines TNF-α, IL-1β, and IL-6 in SARS-CoV-2 positive (*n* = 40) and SARS-CoV-2 negative subjects (*n* = 40). Significantly increased serum levels were observed for all cytokines (TNF-α (**A**), IL-1β (**B**), and IL-6 (**C**)) (*p* < 0.001 for all comparisons). White squares indicate SARS-CoV-2 negative values, and red circles indicate SARS-CoV-2 positive values. All data are shown as means ± SD. Statistical differences between means were evaluated using the unpaired *t*-test with Welch’s correction. *** *p* < 0.001 vs. “SARS-CoV-2 negative” group.

Several weeks following infection, subjects with a history of COVID-19 positivity displayed an increased expression of all the above-mentioned cytokines compared to control groups (Figure 2A–C).

### 2.3. Evaluation of PTX3 and TLR4/NF-κB Axis, Stratifying SARS-CoV-2 Positive Subjects and Non-Infected Control Individuals by Gender

Specific gender differences in the immune response associated with SARS-CoV-2 infection have been previously reported [34,35]; however, literature studies have poorly considered gender variability related to PTX3 expression.

Thus, we stratified the previously described comparisons of PTX3 serum levels and TLR4/NF-κB pathway according to gender. 

**Figure 3:**
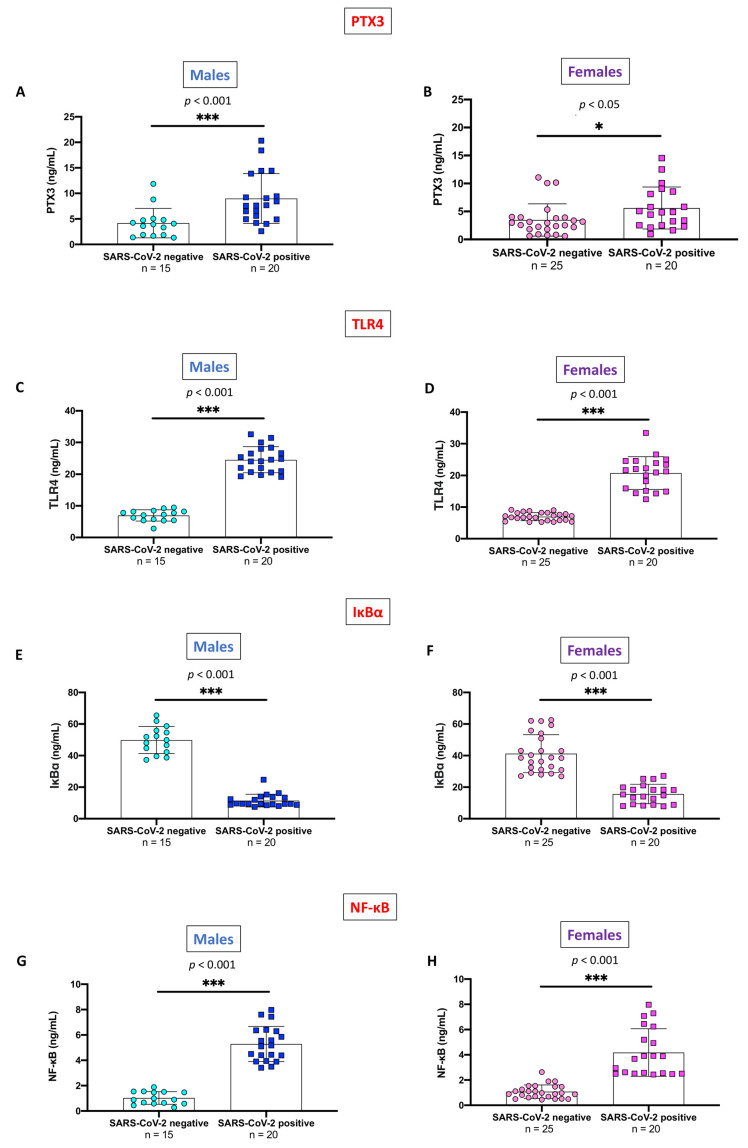
Gender-stratified analysis of PTX3, TLR4, IκB-α, and NF-κB, in SARS-CoV-2 positive subjects and never-infected individuals. PTX3 ((**A**) Males; (**B**) Females), the above difference was more pronounced in males (*p* < 0.001) than in the female counterpart (*p* < 0.05). TLR4 ((**C**) Males; (**D**) Females), IκB-α ((**E**) Males; (**F**) Females), and NF-κB ((**G**) Males; (**H**) Females). Statistically significant differences were found in the comparison between SARS-CoV-2 positive and SARS-CoV-2 negative subjects regarding TLR4, IκB-α, and NF-κB in both genders (*p* < 0.001 for all comparisons). Blue circles indicate values of SARS-CoV-2 negative males while blue squares indicate values of SARS-CoV-2 positive males. Pink circles indicate values of SARS-CoV-2 negative females while pink squares indicate values of SARS-CoV-2 positive females. All data are shown as means ± SD. The unpaired *t*-test with Welch’s correction was used to calculate statistical differences between means. *** *p* < 0.001 vs. “SARS-CoV-2 negative”; * *p* < 0.05 vs. “SARS-CoV-2 negative”.

Serum PTX3 levels were significantly higher in either gender SARS-CoV-2 positive subjects than in healthy individuals; however, the statistical difference was more pronounced in males (*p* < 0.001) (Figure 3A) than in females (Figure 3B) (*p* < 0.05).

Likewise, relevant gender differences between SARS-CoV-2 positive and SARS-CoV-2 negative subjects were also confirmed for TLR4 (Figure 3C,D), IκB-α (Figure 3E,F), and NF-κB (Figure 3G,H) (*p* < 0.001 for all these comparisons).

### 2.4. Evaluation of Pro-Inflammatory Cytokines Stratifying SARS-CoV-2 Positive Subjects and Non-Infected Control Individuals by Gender

Previous studies have shown variations in pro-inflammatory cytokines blood levels between the two genders during the acute phase of the disease [36].

**Figure 4:**
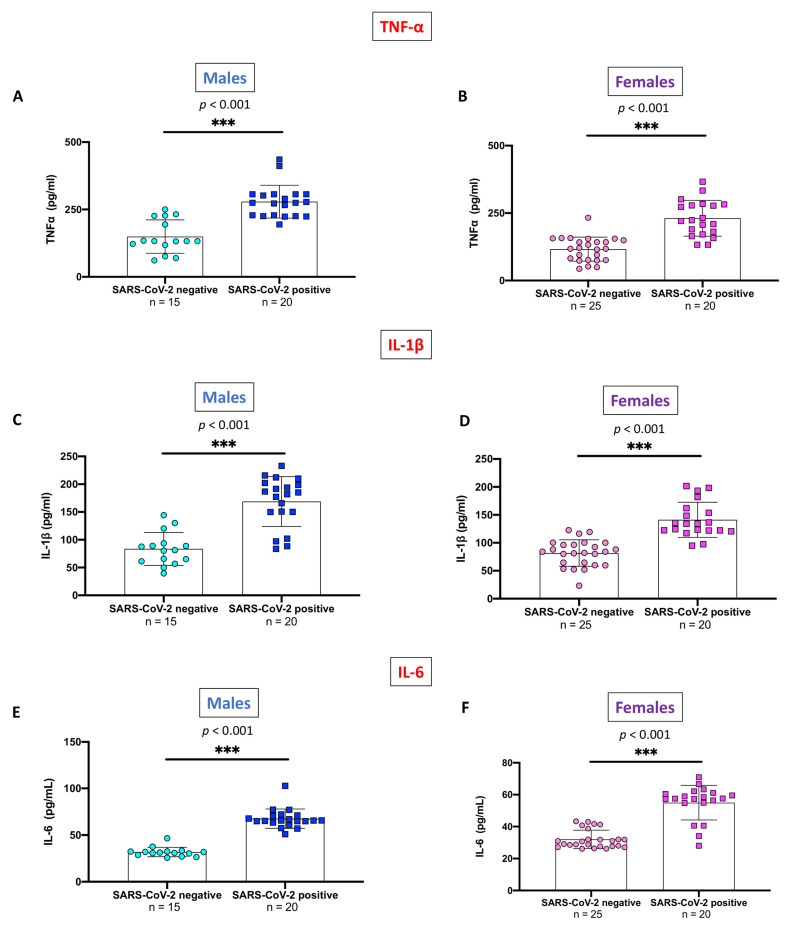
Gender-stratified analysis of pro-inflammatory cytokines serum levels between SARS-CoV-2 positive and SARS-CoV-2 negative subjects. Significant differences between groups in TNF-α ((**A**) Males; (**B**) Females), IL-1β ((**C**) Males; (**D**) Females), and IL-6 ((**E**) Males; (**F**) Females) serum levels were observed (*p* < 0.001 for all comparisons). Blue circles indicate values of SARS-CoV-2 negative males while blue squares indicate values of SARS-CoV-2 positive males. Pink circles indicate values of SARS-CoV-2 negative females while pink squares indicate values of SARS-CoV-2 positive females. All data are shown as means ± SD. Statistical differences between means were calculated using the unpaired *t*-test with Welch’s correction. *** *p* < 0.001 vs. “SARS-CoV-2 negative” group.

On this basis, gender-stratified differences in the pro-inflammatory cytokines TNF-α, IL-1β, and IL-6 were also explored. If compared to SARS-CoV-2 negative, both male and female SARS-CoV-2 positive subjects displayed significantly higher serum levels of TNF-α (Figure 4A,B), IL-1β (Figure 4C,D), and IL-6 (Figure 4E,F) (*p* < 0.001 for all the comparisons).

### 2.5. Evaluation of Pro-Inflammatory Markers Stratifying SARS-CoV-2 Positive Subjects by Gender

**Figure 5:**
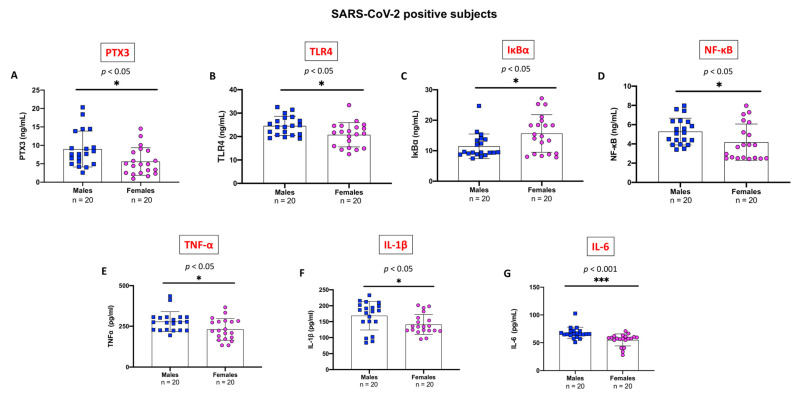
Evaluation of gender-specific differences in SARS-CoV-2 positive subjects. Subgroup analysis has shown that SARS-CoV-2 induced a stronger long-term inflammatory response in males with a previous history of SARS-CoV-2 infection compared to females. Significantly higher levels of PTX3 (**A**), TLR4 (**B**), NF-κB (**D**), TNF-α (**E**), IL-1β (**F**), and IL-6 (**G**) were found in males than females. However, levels of IκB-α (**C**) were significantly lower in males in comparison to females. Blue squares indicate values of SARS-CoV-2 positive males while pink circles indicate values of SARS-CoV-2 positive females All data are shown as means ± SD. Statistical significance between means was evaluated using the unpaired *t*-test with Welch’s correction. *** *p* < 0.001 vs. “Males” group; * *p* < 0.05 vs. “Males” group.

Finally, for each of the above-mentioned inflammatory markers, we compared serum levels between SARS-CoV-2 positive male and female subjects. Interestingly, significant differences between males and females were found for each of the studied inflammatory markers. Higher mean serum levels of PTX3 (Figure 5A), TLR-4 (Figure 5B), NF-κB (Figure 5D), TNF-α (Figure 5E) IL-1β (Figure 5F), and IL-6 (Figure 5G) were observed in male subjects compared to the female counterpart. Notably, IL-6 levels were more pronouncedly higher in males than in females (*p* < 0.001).

On the other hand, IκB-α levels were significantly lower in SARS-CoV-2 positive males compared to the female group (*p* < 0.05) (Figure 5C).

## 3. Discussion

SARS-CoV-2 is a coronavirus belonging to the Coronaviridae family and Coronavirinae sub-family [37], initially identified in late 2019 in Wuhan and later spread wildly infecting millions of people worldwide [38].

The rapid viral replication in the alveolar cells causes an aberrant inflammatory response that leads to a Th1 response, a massive infiltration of macrophages and neutrophils into the lung tissue, the release of pro-inflammatory cytokines, and other severe clinical complications [39]. This pathogenic phenomenon, known as “cytokine storm”, causes serious and, occasionally, fatal extrapulmonary and pulmonary consequences that could result in multiorgan failure [40]. According to these pathogenic assumptions, a persistent inflammatory state accompanied by cytokine hypersecretion may be to blame for a condition of mild latent inflammation known as SIR, which could result in symptoms of chronic inflammation several months following COVID-19 infection [41].

Moreover, other biological processes connected to post-COVID-19 infection were also recently clarified. First, it has been proposed that the pathophysiology of protracted COVID-19 may be influenced by virus-driven cellular modifications and its neurotropism [41]. This mechanism could explain olfactory abnormalities or autonomic nervous system dysfunction [41]. Succeeding, it has been assumed that the immune response, triggered by original infection or hidden viral persistence, may result in harmful conditions such as autoimmune symptoms, activation of the coagulation and fibrosis pathways, or metabolic issues [41]. Importantly, patient-based empirical research raised the notion that autoimmune or inflammatory processes might lead to organ damage [41]. In addition, researchers detected viral particles in numerous organs following the acute infection, thus hypothesizing permanent and occult virus presence [41]. Hence, it is likely that many interrelated pathophysiological pathways could contribute to the clinical picture of chronic COVID-19.

Therefore, the in-depth knowledge of immune-inflammatory pathways as well as the discovery of new molecular targets and innovative care is an important goal for research and management of SIR in COVID-19 patients.

In this framework, pentraxins seem to be promising candidates, since they are proteins involved in complement activation and amplification via communication with complement initiation pattern recognition molecules [27]. In particular, PTX3 can be synthesized by macrophages, monocytes, leukocytes, dendritic cells, adipocytes, endothelial cells, and smooth muscle cells [23], and after its release, it can be recognized by several viruses like cytomegalovirus [42], influenza [43], and members of the coronavirus family, including SARS-CoV-2 [44,45].

Concerning this, previous research has shown that PTX3 levels are a reliable indicator of death in COVID-19 patients. Indeed, Brunetta et al. noted that not-surviving COVID-19 ICU patients had considerably greater PTX3 levels than patients admitted to wards or patients who survived [18]. They also discovered that PTX3 levels were substantially related to laboratory parameters, concluding that this protein is a crucial predictor of mortality, compared to other inflammatory indicators like IL-6 and CRP [18].

Likewise, PTX3 was also promoted as a significant predictor of 28-day ICU mortality by Gutmann et al. in 2021 [46], and later by Hansen et al. in 2022. These studies indicated PTX3 as a useful clinical biomarker for predicting respiratory failure and risk of death at 30 days in COVID-19 patients treated with or without remdesivir and dexamethasone [47]. Conversely, little has been clarified on the PTX3 systemic trend in a follow-up longer than 30 days. Thus, considering the possible role of PTX3 in the immunopathology of SIR forms related to COVID-19, we investigated its influence on the inflammatory course in confirmed SARS-CoV-2 positive health workers compared to never-infected individuals.

In the present study, the obtained data revealed a higher expression of PTX3 in previously infected COVID-19 individuals compared to never-infected subjects, confirming the potential key role of this protein in driving the immune-inflammatory process. Furthermore, stratifying subjects according to gender, a greater statistical significance was found in men than in women compared to the respective controls. This evidence was also validated by a direct comparison of COVID-19 subjects, in which the male component showed significantly higher serum levels of PTX3 than females. Our results are in line with previous studies which highlighted that males tend to experience a more serious course of COVID-19 and related worse outcomes than females [48,49].

TLRs, RIG-like receptors (RLRs), NOD-like receptors (NLRs), and absent in melanoma 2 (AIM2)-like receptors (ALRs) are a set of extracellular and cytosolic receptors present in immune cells, and that enable quick inflammatory signals following interaction with pathogen-associated molecular patterns (PAMPs) and damage-associated molecular patterns (DAMPs) [50]. In particular, in several pathological settings, it was observed that the glycoprotein PTX3 functions through a TLR4-dependent pathway [28], which was up-regulated following tissue damage both locally and in the blood [26].

In accordance, herein we found a considerable overexpression of systemic TLR4 levels in subjects with a history of SARS-CoV-2 positivity compared to SARS-CoV-2 negative, thus demonstrating a consistent immune response following COVID-19 infection. Furthermore, serum TLR4 concentrations increased in previously infected COVID-19-positive men than women; these data confirm that PTX3 was strongly linked with the hyperactivation of the TLR4 pathway.

TLR4 activation sets off downstream stimulation of NF-κB, also favoring in turn, the degradation of IκBα [51]. IκBα breakdown is a key step in the release of the NF-κB component, which then translocates to the nucleus triggering gene transcription and inducing the expression of numerous pro-inflammatory genes [52].

Moreover, literature data indicated the existence of a direct role of PTX3 in the modulation of the TLR4/NF-kB pathway. In this regard, it has been previously shown by Rathore and colleagues that PTX3 silencing significantly reduced the expression of the whole TLR4/NF-kB axis. Consistently with these observations, in the same study, the authors indicated reduced biological effects of PTX3 after inhibiting different molecules of the downstream pathway [28]. As a further example, more recently, in an in vivo model of inflammatory pain, it has been shown that PTX3 silencing can reduce LPS-induced inflammation by directly down-regulating the TLR4/NF-κB signaling pathway [53].

Likewise, the results obtained from this study confirmed a notable increase in NF-κB levels and a significant degradation of IκBα among previously COVID-19 positive population compared to individuals never infected. As well, NF-κB activity was more marked in previously COVID-19-positive males than females.

The activation of NF-κB results in the production of a variety of inflammatory cytokines and chemokines, and this process appears to be especially interrelated to an inadequate immune response during illnesses [54].

Of interest, numerous investigations have demonstrated that both moderate and severe COVID-19 instances may cause a hyperinflammatory reaction with increased levels of many cytokines such as IL-6, TNF-α, etc. [55]. Although the production of these pro-inflammatory species is expected in the early phase of viral infections, SARS-CoV-2 encodes numerous proteins that specifically avoid the type I interferon response, as a result, cytokines’ response is protracted and, in some instances, dysregulated [56].

In confirmation of these hypotheses, clinical studies on long-term consequences in COVID-19 patients have revealed an aberrant diffuse inflammatory cytokine profile and consequent uncontrolled systemic inflammatory response, known as cytokine storm, that lasts for at least 8 months in these individuals [13,55].

In agreement with previous scientific studies, our results supported the upstream of cytokines in subjects with a history of COVID-19 positivity regarding the COVID-19-negative group. These data were further sustained by subgroup analysis in which previously COVID-19-positive males revealed greater cytokines levels, especially in IL-6 if compared to enrolled positive females.

Taken as a whole, results obtained from the present study corroborate altered inflammatory response following SARS-CoV-2 infection in which PTX3 could have a key role, driving PTX3-driven immunoinflammatory cross-talks and leading to a systemic hyperinflammatory state. These outcomes are consistent with the latest scientific evidence in the field of SIR associated with COVID-19 infection, in which inflammatory markers increased until 28 days post-infection [57]. Otherwise, our study indicated PTX3 as a credible inflammatory marker even in a follow-up of more than one month after infection, thus opening a new perspective on the validity of this protein in the context of COVID-19-related systemic inflammation. Furthermore, we support the development of PTX3-targeting drug therapies as a promising approach to moderate inflammatory response in COVID-19 patients or other clinical settings.

Nevertheless, despite the intriguing results, some study limitations must be addressed. First, the size of subjects available for study was small. Moreover, the use of a single method such as ELISA, as well as the evaluation of the parameters only at a systemic level, did not allow for an all-round evaluation. Therefore, it remains to be clarified in future studies whether the local expression of PTX3 also varies in highly affected organs during acute phase and long-term COVID-19 infection. Considering this, future studies will be able to better validate these preliminary findings, thus providing a more robust characterization regarding the immunomodulatory role of PTX3 and its driven signaling pathways in different COVID-19 clinical pictures.

## 4. Materials and Methods

### 4.1. Enrolled Subjects

A case–control study was conducted on healthcare professionals of the AOU Policlinic “G. Martino” in Messina which, following the third dose of the Comirnaty vaccine (Pfizer/BioNTech, New York, NY, USA) in November 2021, voluntarily performed a serological screening as part of hospital monitoring. The sample was split into two groups: vaccinated participants without a history of swab positivity (never-infected subjects), and vaccinated subjects with a history of swab and serological positivity, for a total of 80 individuals.

Individuals’ characteristics are described in Table 1. A higher prevalence of female sex was observed, with a total mean age of 40.9 ± 11.6 years for all subjects included. No other comorbidity or long COVID-19 symptoms were described by the subjects of this study.

### 4.2. ELISA Kit

PTX3 (#EH386RB; assay range: 0.08–20 ng/mL), TLR-4 (#EH460RB; assay range: 0.4–100 ng/mL), IκBα (#EH253RB; assay range: 0.65–150 ng/mL), NF-κB (#MBS260718; assay range: 0.156–10 ng/mL), TNF-α (#BMS223INST; assay range: 7.8–500 pg/mL), IL-1β (#BMS224-2; assay range: 3.9–250 pg/mL) and IL-6 (#BMS213-2; assay range: 1.56–100 pg/mL) ELISA kits were used to measure the levels of each marker in human serum. The ELISA kits were performed in accordance with the manufacturer’s instructions as previously described [58] and briefly reported below.

First, we diluted the standard solution to produce a dilution series to obtain a standard curve. Then we added 100 μL of respective standards to the appropriate wells while in other wells we added 100 μL of diluted samples. Subsequently, the plate was covered and incubated for 2.5 h at room temperature with gentle shaking.

After discarding the solution and washing four times with 1X Wash Buffer, we added 100 μL of prepared biotin conjugate to each well and we incubated the plate for 1 h at room temperature with gentle shaking. The discard and four times washing were repeated. Then, 100 μL of prepared Streptavidin-HRP solution was added to each well, and the plate was once again incubated for 45 min at room temperature with gentle shaking. The discard and four times washing were repeated. By adding 100 μL of TMB substrate to each well, the substrate began to turn blue. In this step, we incubated the plate for 30 min at room temperature in the dark with gentle shaking.

Moreover, in each plate of ELISA analyses, we included negative control samples to validate our results and to check for non-specific binding as well as false positive results. Finally, we added 50 μL of Stop Solution to each well, the solution in the well changed from blue to yellow, then, we read the absorbance at 450 nm using a microplate reader (Thermo Scientific™ Multiskan™ FC Microplate Photometer, Waltham, MA, USA).

### 4.3. Statistical Analysis

The values are expressed as the mean ± standard deviation (SD) of N observations, each N represents the number of subjects studied. All the results were analyzed using the unpaired *t*-test with Welch’s correction; only a *p*-value of less than 0.05 was considered significant.

## 5. Conclusions

Taken together, the data discussed and examined in this study highlighted the potential of PTX3 as a reliable biomarker in predicting inflammatory systemic alterations following COVID-19 infection, providing an innovative perspective on the usefulness of this protein in the SIR pathological context. Analyzing PTX3 and related panels of inflammatory proteins, inflammatory overload appears to be consistent in both previously COVID-19-positive men and women, resulting statically more significant in the male population. As far as we know, this is the first study relating PTX3 with gender differences in the COVID-19 clinical picture.

Thus, these intriguing findings can constitute an important add-on in the discovery of novel biomarkers best-fitted to COVID-19 pathophysiology and consequences. Likewise, Karimi and colleagues advised regarding the usefulness of 11 groups of systemic inflammatory markers for risk-stratifying patients and prognosticating outcomes related to COVID-19 [59]. Those markers included neutrophil to lymphocyte ratio (NLR), derived NLR (d-NLR), platelet to lymphocyte ratio (PLR), lymphocyte to monocyte ratio (LMR), and lymphocyte to CRP ratio (LCR) among others, which index can be obtained through routinary and widely available laboratory tests [59].

As well, we also retain that the assessment of PTX3 by laboratory testing should be strongly advised by clinicians given its significance in triggering the immunoinflammatory responses. Moreover, considering our findings, we also hypothesize that the block of the PTX3/TLR4/NF-κB pathway could be a potential strategy to control the inflammatory response in several human diseases.

## Figures and Tables

**Table 1:** Characterization of enrolled healthcare workers.

	Total Subjects		SARS-CoV-2 Positive		Never-Infected Subjects	
Sex	35 (M) 45 (F)		20 (M) 20 (F)		15 (M) 25 (F)	
Age	40.9 ± 11.6		39.5 ± 10.8		43.0 ± 13.1	
	Males	Females	Males	Females	Males	Females
	40.2 ± 12.0	41.5 ± 11.7	39.7 ± 13.0	39.4 ± 8.5	41.5 ± 11.0	43.9 ± 14.9
Comorbidities	None		None		None	
Months from COVID-19 positivity	6.24 ± 3.51		Males	Females	/	
			5.00 ± 2.55	7.63 ± 4.07		

## Data Availability

All the results were included in this study and available to the corresponding author’s address.
